# Faricimab for treatment-resistant choroidal neovascularization (CNV) in neovascular age-related macular degeneration (nAMD): seven-months results using artificial intelligence and OCTA

**DOI:** 10.1186/s40942-025-00691-4

**Published:** 2025-06-17

**Authors:** Anna Heinke, Alexandra Warter, Ines D. Nagel, Akshay Agnihotri, Nehal Nailesh Mehta, Carlo Miguel B. Galang, Daniel N. Deussen, Dirk-Uwe G. Bartsch, Lingyun Cheng, Henry A. Ferreyra, William R. Freeman

**Affiliations:** 1 Jacobs Retina Center, 9415 Campus Point Drive, La Jolla, San Diego, CA 92037 USA; 2 Viterbi Family Department of Ophthalmology and Shiley Eye Institute, University of California San Diego, 9415 Campus Point Drive, La Jolla, San Diego, CA 92037 USA; 3 Division of Ophthalmology Informatics and Data Science, Viterbi Family Department of Ophthalmology and Shiley Eye Institute, University of California San Diego, 9415 Campus Point Drive, La Jolla, San Diego, CA 92037 USA; 4 Department of Ophthalmology, University Hospital, Ludwig-Maximilians-University, 80336 Munich, Germany; 5 Department of Ophthalmology, University Hospital Augsburg, 86156 Augsburg, Germany

**Keywords:** ANG-2 (Angiopoietin 2), Anti-VEGF (Anti- Vascular Endothelial Growth Factor), Faricimab (VABYSMO) or faricimab-svoa, Intraretinal fluid (IRF), Subretinal fluid (SRF), PED (Pigment Epithelium Detachment), Wet AMD (Wet Age-Related Macular Degeneration) AMD or ARMD or neovascular age-related macular degeneration (nAMD), Artificial intelligence (AI)

## Abstract

**Background:**

To analyze the therapeutic response to faricimab 6 mg/0.05 ml in eyes with neovascular AMD (nAMD) with refractory intra- and/or subretinal fluid due to choroidal neovascularization (CNV), previously unresponsive to 4 mg monthly aflibercept and combination therapy with anti-VEGF and long-acting steroids.

**Methods:**

A retrospective case series study of 22 eyes with unresponsive CNV, despite monthly intravitreal treatment (mean number of pre-faricimab injections: 35.52 ± 17.12). We evaluated therapeutic response in eyes with persistent intra/subretinal fluid (IRF/SRF) unresponsive to anti-VEGF double-dose (DD) monotherapy (4-mg aflibercept) and/or simultaneous DD anti-VEGF (4-mg aflibercept) with steroids (triamcinolone). Best-corrected visual acuity (BCVA), intraocular pressure (IOP), and optical coherence tomography (OCT) measurements of central retinal thickness (CRT) were recorded for 7 follow-ups. Baseline and follow-up OCTs were examined by an AI-developed platform (Discovery OCT Fluid and Biomarker Detector, RetinAI AG, Switzerland) to measure the volume of IRF, SRF, and pigment epithelium detachment (PED) in nanoliters (nL) and CRT in micrometers (μm). Paired t-test compared these parameters at baseline and after treatment. OCTA analysis of CNV before and after treatment with faricimab was conducted using Angio-Tool software.

**Results:**

Anatomic outcomes included mean CRT reduction of -25.3 μm (p = 0.0118) at month-1, -16.15 μm (p = 0.0414) at month-4, and -26.36 μm (p = 0.0129) after the 7th follow-up. AI-assisted software analysis showed a significant reduction of IRF, SRF, and PED volume at multiple time points after initiating faricimab. There was a non-significant improvement in BCVA.

**Conclusions:**

Switching to faricimab improved anatomy in highly treatment-resistant CNV eyes, indicating its potential when other therapeutic options have failed.

## Introduction

Neovascular age-related macular degeneration (nAMD) affects nearly 11 million patients in the United States and is considered a leading cause of irreversible blindness in developing countries [1]. Patients progressively lose central vision due to intraretinal and/or subretinal fluid seen in Optical Coherence Tomography (OCT, 1). The progression of AMD is linked to the imbalance of primarily vascular endothelial growth factor A (VEGF A) and less frequently other growth factors and cytokines, leading to choroidal neovascularization (CNV), which breaches the subretinal pigment epithelium, causing disease progression and treatment resistance [1, 2].

Initial randomized phase III clinical trials, such as MARINA and ANCHOR, established monthly intravitreal anti-VEGF injections as the gold standard for the treatment of neovascular age-related macular degeneration [nAMD, 3, 4]. Despite these advances, some AMD cases remain unresponsive to monthly anti-VEGF treatment. Combination therapies, including photodynamic therapy (PDT) and intravitreal steroids with anti-VEGF agents, have been explored to enhance treatment outcomes [5–8]. However, neovascular AMD unresponsive to monotherapy and combination therapy remains challenging, necessitating novel approaches.

Current FDA-approved treatment for wet age-related macular degeneration consists of anti-VEGF agents such as ranibizumab (Lucentis, Genentech, San Francisco, CA, USA), aflibercept (Eylea, Regeneron, Tarrytown, NY, USA), brolucizumab (Beovu, Novartis, East Hanover, NJ, USA, currently withdrawn from the US market), Eylea-HD (Regeneron Pharmaceuticals, Inc.) and biosimilars. In early 2022, the FDA approved the intravitreal injection of faricimab (Vabysmo, Genentech, South San Francisco, CA, USA), a novel bispecific monoclonal antibody that simultaneously targets 2 key pathways: VEGF and angiopoietin-2 (Ang-2), for treatment-naive AMD and Diabetic Macular Edema [9] and later for Retinal Vein Occlusion (RVO, 10). Clinical trials, such as AVENUE and STAIRWAY, have shown faricimab's non-inferiority to standard anti-VEGF therapy (ranibizumab), with extended treatment intervals of 12–16 weeks after a loading period of every 4 weeks for 4 months [9]. Subsequent trials, TENAYA and LUCERNE, demonstrated its safety and efficacy, allowing extended dosing intervals compared to aflibercept [10].

Faricimab's dual mechanism of action is hypothesized to provide greater efficacy and durability in treating nAMD compared to traditional anti-VEGF agents.

Recent data from the TRUCKEE study, which evaluated clinical visual and anatomic outcomes in patients with nAMD who were either treatment-naive or previously treated, showed that after a single injection of faricimab, there was a + 1.1 letter improvement in best-corrected visual acuity (P = 0.035) and a 31.1-micron reduction in central subfield thickness (P < 0.001) in all eyes (n = 337). Similar results were observed after three injections in all eyes (n = 94, 11).

Other recent real-world studies demonstrated the efficacy of treatment with faricimab injections in previously treated patients by improvement or maintenance of visual acuity for patients with nAMD, along with rapid improvement of anatomical parameters [12, 13].

This study examines the efficacy of faricimab in highly treatment-resistant nAMD cases, specifically those unresponsive to monthly 4 mg aflibercept (double of the 2 mg standard dose, 4 mg/0.1 cc) and combined anti-VEGF and steroid therapies. The unique population and treatment protocol in this study aims to address the need for longer-acting agents and better disease management in these difficult cases. To assess that we analyzed the response of fluid reduction in volume using AI-assisted software and explored the quantitative OCTA vessel changes.

## Methods

This is a retrospective chart review of 22 eyes of 20 consecutive patients with nAMD that presented with persistent IRF and/or SRF despite 4 mg of monthly aflibercept (high volume-high frequency, HVHF) anti-VEGF monotherapy (aflibercept 0.1 cc/4 mg) and/or simultaneous anti-VEGF and triamcinolone acetonide IVI (combination) for a minimum of 6 consecutive months prior to receiving faricimab. These patients were subsequently treated with 6 mg (0.05 mL solution) faricimab IVI injection (6.0 mg/0.05 ml, Vabysmo®, Genentech Pharmaceuticals, Inc.) for 6 consecutive months (in the period from April 2022 to March 2023) at the Jacobs Retina Center, University of California San Diego (UCSD) Shiley Eye Institute. Written informed consent was obtained for each patient prior to receiving intravitreal injections for the standard of care procedure. UCSD Institutional Review Board (IRB Nr#120516) approval was acquired for the review and analysis of patient data. All procedures adhered to the tenets of the Declaration of Helsinki for research involving human subjects and complied with Health Insurance Portability and Accountability Act (HIPAA) regulations.

The study's primary aim is to determine the efficacy of faricimab in patients with treatment-resistant IRF and/or SRF as defined above due to neovascular age-related macular degeneration (nAMD). We included unresponsive or partially responsive eyes (n = 22) that presented persistent intraretinal and/or subretinal fluid as seen in OCT. Resistance was defined as non-responsiveness to standard (aflibercept 0.05 cc/2 mg for at least 4 months) and to escalated treatment with high-volume and high-frequency anti-VEGF (aflibercept 0.1 cc/4 mg every 4 weeks, HVHF) and/or combined anti-VEGF and steroids for a minimum of 6 months [8, 14, 15]. A minimum of 4 months of steroid washout was required before initiation of treatment with faricimab. The study was conducted before FDA approval of Eylea HD 8 mg, and we are discussing the off-label use of 4 mg of Eylea (0.1 cc) which is double of standard 2 mg/0.05 cc of Eylea. Our group has previously published the results of using high-dose high-frequency aflibercept (4 mg/0.1 cc) for recalcitrant age-related macular degeneration [14].

All patients unresponsive to intensive therapy as described above for the duration of a minimum of 6 months and with 49-B scan SD-OCT volume scans of the central macula centered on the fovea (Heidelberg Spectralis HRA + OCT Spectral Domain (Heidelberg Engineering, Heidelberg, Germany) with persistent IRF/SRF were included in the study. SD-OCT acquisition protocol consisted of a volume scan (20 × 20-degree map, 49 lines, 768 A-scans per line) and cross-sectional line scans (horizontal and vertical; 30-degree length) with 9-times image averaging. OCTA scans (number of B-scans = 512, pattern size = 3 · 3 mm/10° · 10°, distance between B-Scans = 6 mm, ART images average = 5), centered on the fovea were acquired. Eyes that evidenced response to anti-VEGF and/or combination treatment were excluded (i.e. those that achieved completely dry macula seen in volume 49-B scan SD-OCT scan). Two independent graders (A.H. and A.W.) reviewed images qualitatively to determine the presence/absence of IRF and SRF. Any disagreements were adjudicated by a third senior grader (W.R.F). No quantitative threshold was applied for the presence of retinal fluid. Patients who had other causes of choroidal neovascularization (e.g., trauma, idiopathic, myopic degeneration) were excluded from the study.

Baseline characteristics of patients were obtained through a review of electronic medical records (EMR). Patients’ age, gender, mean number of intravitreal injections before faricimab, lens status (pseudophakia vs cataract) were recorded and are summarized in Table 1in the results section. Primary outcome measures included change in best-corrected visual acuity (BCVA) and maximal central retinal thickness (CRT). Secondary outcomes include change in volume of SRF/IRF and PED volume measured in nanoliters (nL) with AI-assisted software (Discovery OCT Fluid and Biomarker Detector, RetinAI AG, Switzerland). Automated segmentation and volumetric analysis of IRF, SRF, and PED were performed using the Discovery OCT Fluid and Biomarker Detector,a CE-marked AI-powered image analysis platform. No model training or independent validation of the algorithm was performed as the software was used as a proprietary clinical research tool. Only the raw quantitative outputs were used for analysis. The baseline measurements were considered the last OCT and BCVA obtained prior to intervention with faricimab. Complications as a result of faricimab injection (e.g., retinal pigment epithelium (RPE) tear, intraocular inflammation) were recorded.

**Table 1:** Baseline characteristics, n = 22 eyes. 20 patients

Age	Gender Total N of Patients = 21	Pseudophakia/Phakia	Number of intravitreal injections	BCVA (logMAR)	IOP (mmHg)	Max RT (μm)	IRF volume (nL)	SRF volume (nL)	PED volume (nL)
Mean 81.60, ± SD 7.88	Male = 9 Female = 11	20/22 (90%)	Mean 34.82 ± SD 17.10	Mean 0.52 logMAR ± SD 0.43, Snellen 20.70 + 1	Mean 12.3 ± SD 3.30	Mean 387.45 ± SD57.58	Mean 22.55 ± SD27.37	Mean 20.9 ± SD32.4	Mean 164.7 ± SD200.67

Early Treatment Diabetic Retinopathy Study (standardized retro-illuminated ETDRS chart, Good-Lite) BCVA testing at 4 m was recorded during the exam in the clinic and the LogMAR value of the vision was used for statistical analysis. Each visit consisted of a slit-lamp (Haag-Streit, USA), intraocular pressure (IOP, Goldmann tonometer or iCare tonometer- Icare, USA, Inc.), and dilated ophthalmic examination.

### Quantitative analysis: artificial intelligence–based biomarker optical coherence tomography detector

**Fig. 1:**
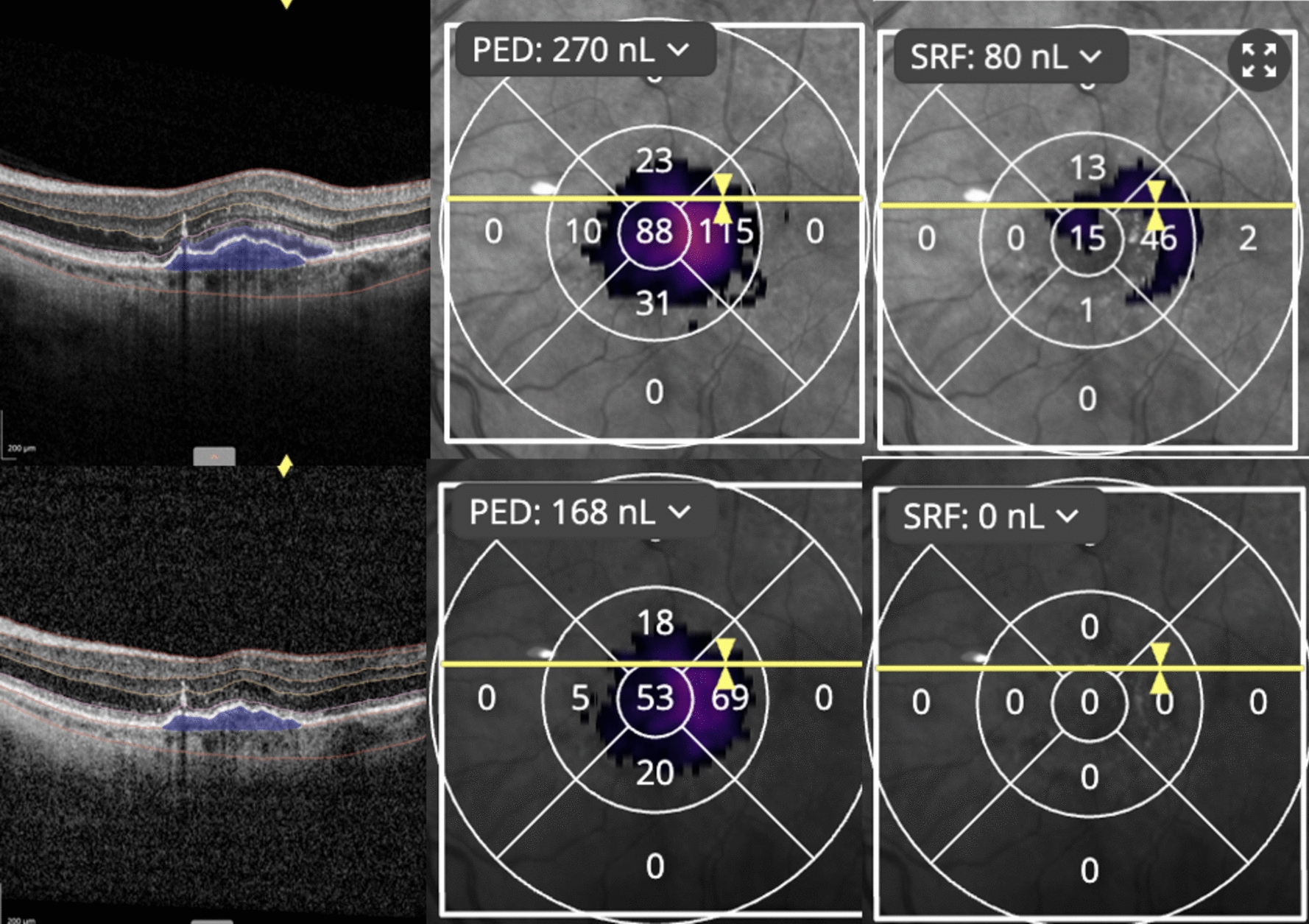
The top half shows the patient with resistant CNV pre-faricimab treatment. The ETDRS zone with the PED in maximum affected zone (MAZ) volume is 115 nL (nanoliters) marked with arrows and the SRF in maximum affected zone (MAZ) is 46 nL (nanoliters). The lower half of the figure shows the same patient 2 months after treatment with faricimab with the reduction of PED in maximum affected zone (MAZ) volume to 69 nL (nanoliters) and SRF in maximum affected zone (MAZ) volume reduction to 0 nL (nanoliters)

**Fig. 2:**
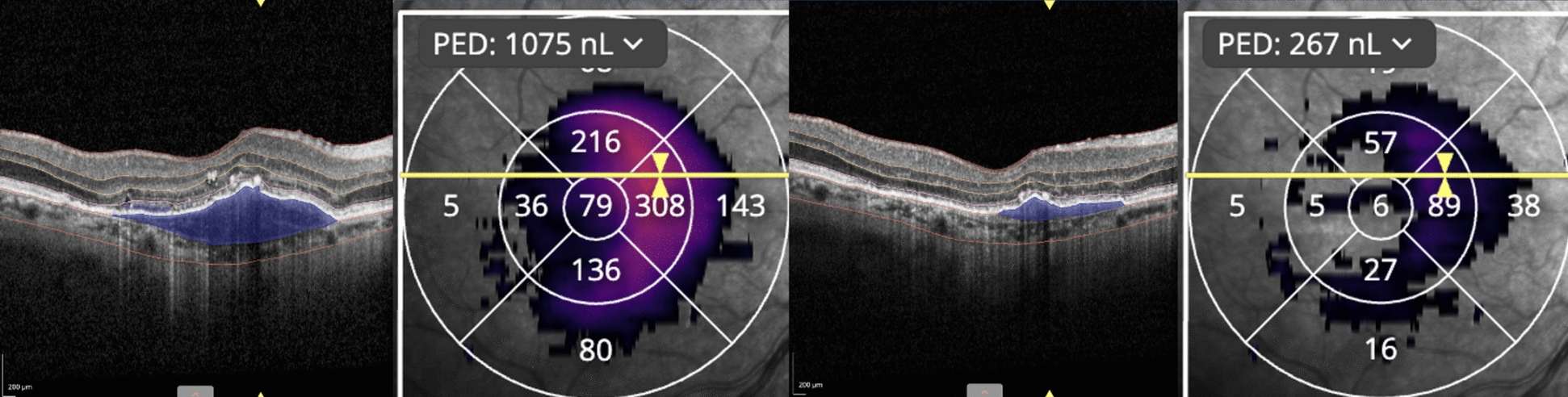
The left hand side image shows the patient with resistant CNV pre-faricimab treatment. The ETDRS zone with PED in the maximum affected zone (MAZ) volume is 308 nL. The right-hand side of the figure shows the same patient one month after treatment with farcimab with the reduction of PED volume in MAZ to 89 nL at the most affected ETDRS zone

We analyzed OCT images using the Discovery OCT Biomarker Detector platform (RetinAI AG, Switzerland). This AI-assisted program automatically quantifies retinal and choroidal thickness, volumes, and biomarkers for each OCT B-scan image [16, 17]. The software performs automated segmentation of retinal and choroidal thickness measurement of several layers: the retinal nerve fiber layer, ganglion cell layer and inner plexiform layer (GCL + IPL), inner nuclear layer and outer plexiform layer (INL + OPL), outer nuclear layer (ONL), photoreceptor and RPE layers (PR + RPE), and the choriocapillaris with choroidal stroma. It also measures the overall retina thickness (RT). For volumes, the program quantitatively measures subretinal fluid (SRF), intraretinal fluid, and pigment epithelium detachment. We have measured the total central retinal thickness (CRT), intraretinal fluid volume (IRF) in nanoliters (nL), subretinal fluid volume (SRF) in nL, and PED volume in nL at each time point. For the statistical analysis, we took the maximum retinal thickness at the central macula (CRT), and we measured and compared the baseline fluid volume (for IRF, SRF, and PED) at the maximum affected zone (MAZ) in the ETDRS grid and we compared it with the same maximum affected ETDRS zone for the consequents follow-up visits to monitor the fluid change (Fig. 1). In a few cases, if the fluid shifted from MAZ to the neighbor ETDRS zone, we summed up the fluid volume from the MAZ and the ETDRS zone where the fluid shifted and used the average volume for the statistical analysis. Figure 1 and Fig. 2 present the case example of how the fluid volume was measured by the software and it illustrates the ETDRS zones with quantified fluid volumes (Fig. 1 and Fig. 2). The measurements were done for seven consecutive visits.

### OCTA CNV analysis with Angio-tool software

Using Angio-tool software (v0.6a, National Cancer Institute, Center for Cancer Research, Bethesda, USA), we measured quantitative parameters of the membrane before faricimab injection and after loading phase (between 3–4 injections, depending on if the OCTA image was available). In our analysis we included the following parameters: vessel area, branching index/junctional density, and lacunarity of the CNV vessel network. We used the same setting for each patient regarding threshold, vessel thickness, and removal of small particles. The Angio tool in a semi-automatic manner, analyzes the explant area (mm^2^), which is the total area after cropping of the CNV complex (CNV lesion size cropped manually with Image J by an experienced retina specialist (A.A)).

Statistical Analysis: The primary outcome was the change in the max central retinal thickness (CRT) following the initiation of faricimab intravitreal injection and the change of BCVA. The secondary outcome was the change in IRF, SRF, and PED volume. Intraocular pressure (IOP) were also recorded prior to faricimab intervention and at subsequent monthly follow-ups for 7 months. Baseline (prior to farcimab) CRT, IRF, SRF, PED volume in the maximally affected zone, logMar of BCVA, and IOP were compared with the corresponding measurement at each follow-up using paired t-test or sign test according to the distribution of the data. All data were analyzed with SAS JMP version 16. A P value < 0.05 was considered statistically significant.

## Results

This study included 20 patients whose baseline characteristics are summarized in Table 1**.**

Paired t-tests showed a mean reduction of CRT that was statistically significant at month 1, 2, 3 and 4. CRT reduction was consistent and statistically significant for 7 consecutive follow-ups, except from follow-up 5 and 6 (Table 2). We observed a statistically significant difference in CRT in a subgroup of patients that were extended after the first 3 injections of faricimab treatment versus patients who didn’t get extended and continued q4w treatment. Patients that continued q4w (no extension) had higher CRT compared to patients who achieved treatment extension (mean difference -36.436μm, p=0.0217).

**Table 2:** Summary of the results (mean reduction of CRT, IRF, SRF fluid, and PED volume in the maximally affected zone (MEZ)) in each tested follow-up, calculated as change from baseline

Parameter	Follow-up nr 1	2	3	4	5	6	7
CRT (μm)	− 25.3; p = 0.0118	− 26.1; p = 0.0414	− 18.2; p = 0.0414	− 16.15; p = 0.0414	− 23.28, p = 0.14*	− 31.87, p = 0.6*	− 26.36; p = 0.0129
IRF (nL)	− 10.211; p = 0.0386	− 13.474; p = 0.0386	− 13.0; p = 0.001	− 11.368; p = 0.0117	− 13.1; p = 0.5*	− 12.0; p = 0.75*	− 4.92; p = 0.29*
SRF (nL)	5.68; p = 0.34*	− 13.4; p = 0.34*	− 12.63; p = 0.11*	− 15.42; p = 0.0117	− 12.778; p = 0.0391	− 14.47; p = 0.07*	− 23.14; p = 0.0078
PED (nL)	− 27.74; p = 0.096*	− 17.4; p = 0.064*	− 8.7; p = 0.1435*	− 46.0; p = 0.09*	− 45.0; p = 0.0386	− 17.3; p = 0.2668*	− 18.23; p = 0.5811*

The asterisk * marked not significant p values. Follow-up Visits 1–4 correspond to treatment Months 1–4. Visits 5–7 represent subsequent follow-ups, which may not align directly with monthly intervals due to individualized treatment extensions

We compared PED volume at each follow-up with the volume at baseline using a paired t-test. Mean PED volume reduced from the baseline at each follow-up, but it was significantly reduced at 5th follow-up after the treatment with mean reduction -45nL (p=0.0386) (Table2).

We analyzed IRF volume at each follow-up with the volume at baseline at the maximum affected ETDRS zone (MAZ) using paired t-test. Mean IRF volume reduced significantly from the baseline in the first 3 months of treatment (follow-up 1,2,3), so after loading phase of the drug. At month 5 and 6, and 7, the IRF reduction was observed, but it was not statistically significant (Table2).

SRF volume at each follow-up was compared with the volume at baseline at the maximum affected ETDRS zone using paired t-test. Mean SRF volume reduced from the baseline, but the mean reduction was not statistically significant in the first 3 months of treatment. Mean SRF was reduced significantly at month 4 after the loading phase of monthly faricimab, and also significantly at the 7^th^ follow-up visit (Table 2).

Unlike the anatomical improvement, BCVA, evaluated per Snellen with logMAR conversion showed non-significant changes.

IOP as a primary safety outcome showed no significant change from baseline during follow-ups. No eyes required any interventions due to elevation in IOP. No glaucoma surgery, vascular occlusions, RPE tears occurred from faricimab injection. No cases of endophthalmitis, retinal breaks, retinal detachment, or formation of geographic atrophy (GA) were observed during the study period. In one case we observed a recurrent anterior uveitis which occurred at injection number 5 and again after injection 6; that patient was changed to aflibercept with no further inflammation.

**Fig. 3:**
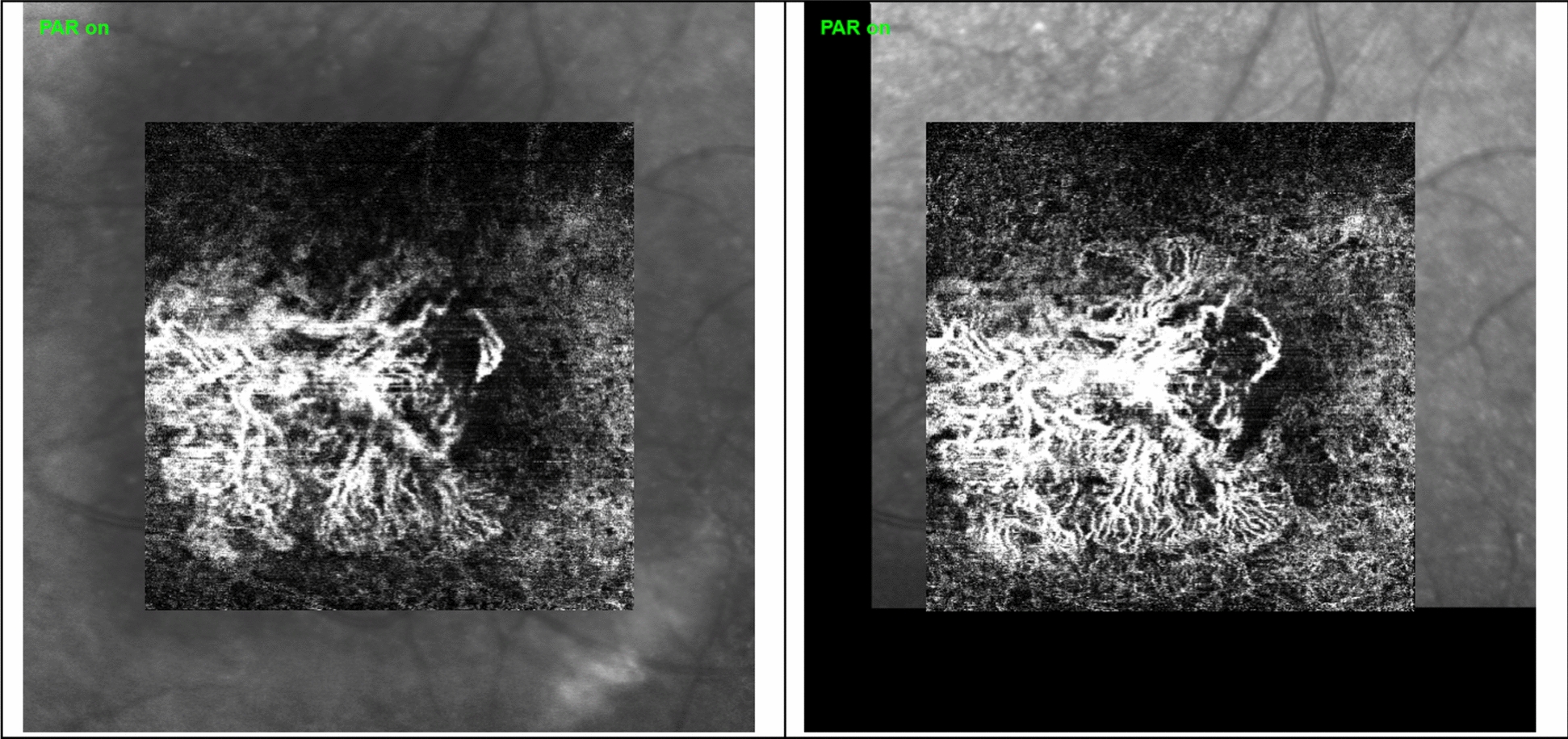
Example of CNV vessels in OCTA (Heidelberg Spectralis) in the avascular zone in one of the study patients before starting faricimab (left-hand-side image) and the image of CNV of the same patient after 3 injections of faricimab (right-hand-side image). The CNV on the right-hand image has better quality and contrast, so vessels are easier to distinguish. However, the mean changes in vascular percentage area, vessel length, junctional density, and lacunarity measured with Angio-Tool software were not statistically significant between these images before and after faricimab treatment

In terms of OCTA CNV vessels analysis with Angio-tool software, none of the measured parameters (vessel percentage area, junctions density, mean lacunarity) showed statistically significant change in paired t-tests. The summary of results measured with Angio-tool are presented in Table 3. We found no difference nor was there a trend of difference in resistant eyes before and after faricimab. An example of OCTA changes before and after faricimab injections in CNV vessels is shown in Figure 3.

**Table 3:** Summary of Angio-tool parameters measured in OCTA images in Avascular Complex (AC) before and after treatment with faricimab. Mean, ± SD, median, and P value

Angio-tool parameter	Pre-faricimab treatment mean and [median] value	Post-faricimab treatment mean and [median] value	P value
Vascular percentage area (%)	22 ± 7 [22]	20 ± 6 [20]	0.22
Branching Index/Junction density	0.0006 ± 0.0004 [0.0004]	0.0005 ± 0.0004 [0.0005]	0.49
Lacunarity	0.38 ± .28 [0.26]	0.41 ± 0.24 [0.36]	0.59

## Discussion

Highly resistant CNVs due to neovascular AMD are difficult-to-treat cases that are both a burden to physicians and patients alike [18], creating an increasing demand for treatments with longer-acting agents that allow for longer intervals while still decreasing disease progression and burden. We and other authors have evaluated alternative treatments with combined steroids and anti-VEGF, with minimal improvement. Other studies have evaluated faricimab and found visual and anatomic improvement, in treatment naïve eyes [10]and previously treated eyes [11, 12], as well as they studied resistant neovascular AMD switched from aflibercept to faricimab [19], but not in those resistant to high-dose monotherapy (aflibercept 4mg/0.1cc) and combination with both anti-VEGF and steroids. Our study is also different from previous ones, because we used the artificial intelligence-assisted software for the detailed fluid analysis in volume (nL) and we have analyzed the OCTA CNV vascular network before and after the treatment with faricimab.

Our population was comprised of eyes highly resistant to both combination and escalated high dose high-frequency anti-VEGF with an average history of 35.52 intravitreal injections. Despite aggressive therapeutic attempts eyes still presented with recalcitrant intra and subretinal fluid. In such eyes, we found overall disease improvement, particularly anatomic with faricimab. BCVA remained stable and had no decline or statistically significant improvement. This absence of statistically significant visual acuity (BCVA) improvement further reinforces the distinction between anatomical and functional outcomes, especially in eyes with long-standing, treatment-resistant disease. Given the chronicity of disease and treatment, we did not expect any change in vision [18] . Our analysis found however, that eyes with a better BCVA at baseline had more improvement regarding CRT reduction in consequent visits. This is likely attributed to the fact that these patients may have not had such an advanced disease or had received treatment sooner [20]. These findings may indicate that earlier use of faricimab should be considered.

Our data showed significant central retinal thickness reduction after 1-st month of faricimab treatment, as well as after 3rd and 4th month and at month 7. Thinning was consistent and significant at each monthly time point, except at month 5 and 6. As expected, patients who needed monthly injections of faricimab had statistically significant higher CRT than patients who were able to be extended, because they didn’t have intra- or subretina fluid in OCT, hence the retina was thinner in this subgroup.

Similarly to Szigiato et al work which studied PED height [21], in our study, we observed the reduction of PED volume although the changes were not statistically significant, except from the 5th month of the analysis. Although PEDs are not a primary target when treating wet-AMD [2, 22], this outcome may be of positive predictive value for treatment extension. In our study group not all the patients had PEDs and with lower number of cases, this parameter showed significance only in one time point. PEDs as a potential biomarker require longer follow-ups and a bigger number of cases to evaluate the value of this outcome.

Significant reductions in IRF volume were observed, at month 1, month 2, month 3, 4 and 7 of the analysis. Some of the time points did not show statistical significance due to the low number of cases (first 3 months of observations). Months 5 and 7 showed a significant mean reduction in SRF. After the loading phase, in case of successful treatment (no fluid IRF and/or SRF fluid in OCT), patients were extended from q4w to q6-q8w decided based on the treating physician’s discretion (W.R.F). Some patients experienced a recurrence of the fluid after treatment extension, hence some fluctuations of fluid volumes and statistical significance were observed in different time points.

The analysis with AI-assisted software allows to monitor subtle changes in retinal biomarkers, such as IRF/SRF and PED volume longitudinally, and assess the outcome of treatment with anti-VEGF. The Angio-tool analysis of vascular parameters has not revealed statistically significant changes in measured vascular parameters in OCTA despite the overall improvement in retinal fluid in OCT. This may be due to the resistant character of the CNV in our studied group. To our knowledge, this hasn’t been studied before in resistant patients treated with faricimab. Future studies should evaluate changes in CNVs vascular parameters in treatment-naive patients treated with faricimab, as the non-treated CNVs may be more responsive to anti-VEGF treatment at the early stage [23, 24].

The limitation of our study is a small sample size which may limit the ability to identify factors associated with the positive response to faricimab. Moreover, while our study provides preliminary insights into the use of faricimab in treatment-resistant CNV, the absence of a control group limits the ability to directly compare outcomes with continued aflibercept therapy. Future prospective studies incorporating a control arm of patients maintained on aflibercept would help clarify the comparative efficacy of faricimab in this patient population. The unique value of our study is the resistant CNV due to wet AMD with clearly defined resistance and consistent treatment protocol and it hasn’t been done in the systematic matter before. Moreover, we have conducted the analysis using artificial intelligence-assisted software that allows a detailed assessment of the fluid biomarkers at the nanoliter level and we conducted the study of CNV vascular parameters using OCTA imaging and Angio-tool software for analysis. While the use of AI-powered fluid quantification software allowed for objective and reproducible assessment of retinal fluid compartments, we acknowledge several limitations associated with its use. Notably, the software employed in this study has not undergone external validation on independent datasets, which may affect the generalizability of the segmentation results. Moreover, standard segmentation performance metrics, such as DICE similarity coefficients, were not available. These factors should be considered when interpreting the results, and future studies incorporating external benchmarking and detailed performance metrics are warranted to validate and refine the current findings.

Faricimab's dual mechanism of action may allow for disease stability in treatment- resistant wet-AMD CNV when all other therapeutic options have been exhausted. The significant change in central retinal thickness and reduction of intra and subretinal fluid with some reduction of PED may infer that earlier use of faricimab may also benefit these treatment-resistant patients [22]. The limitation of this study is the low number of cases. Future studies will be able to evaluate the long-term efficacy of faricimab in the larger groups of these highly resistant patients.

## Data Availability

The datasets generated and/or analyzed during the current study are not publicly available due to the possibility of patients’ identification from retinal imaging but are available from the corresponding author upon reasonable request. For some institutions, this might require a Data Sharing Agreement.

## Abbreviations

ANG-2: Angiopoietin 2
Anti-VEGF: Anti- Vascular Endothelial Growth Factor
IRF: Intraretinal fluid
SRF: Subretinal fluid,
PED: Pigment Epithelium Detachment,
Wet AMD: Wet Age-Related Macular Degeneration
AI: Artificial intelligence
BCVA: Best-corrected visual acuity
CNV: Choroidal neovascularization
OCTA: Optical coherence tomography-angiography
